# Pediatric Primary Cervical Malignant Müllerian Papilloma: A Case Report and Review of Literature

**DOI:** 10.1002/ccr3.72405

**Published:** 2026-05-24

**Authors:** Na Guo, Hui Liu

**Affiliations:** ^1^ The Department of Obstetrics and Gynecology West China Second University Hospital of Sichuan University Chengdu Sichuan China; ^2^ Key Laboratory of Birth Defects and Related Diseases of Women and Children (Sichuan University), Ministry of Education Chengdu Sichuan China

**Keywords:** cervical cancer, child, colposcopic, malignant müllerian papilloma, surgery

## Abstract

In children presenting with prolonged irregular vaginal discharge, colposcopic evaluation should be performed, and the possibility of a malignant tumor must be considered in the differential diagnosis. Surgical excision remains the cornerstone of treatment to prevent recurrence. Long‐term follow‐up is mandatory.

## 1 Introduction

Genital Müllerian papillomas are rare tumors, and all previously reported cases have been benign, with excellent prognosis following local excision [1]. Cervical malignant Müllerian papilloma **is** an exceptionally rare entity, particularly in the pediatric population. We report a case of primary cervical malignant Müllerian papilloma in a 12‐year‐old child, to the best of our knowledge, this represents the first documented case of a malignant Müllerian papilloma arising in the cervix of a prepubertal girl. Herein, we describe our experience with this case and provide a review of the literature, with the aim of contributing to the management of similar rare clinical presentations.

## 2 Case History/Examination

A 12‐year‐old child was hospitalized due to irregular vaginal discharge persisting for more than four months. She had no significant past medical history. Menarche occurred at the age of 11. Transabdominal or pelvic ultrasound revealed a solid cervical mass measuring 6.6 cm × 6.4 cm × 3.5 cm. On gynecological examination, foul‐smelling, necrotic‐like tissue was observed protruding from the vaginal introitus. Positron emission tomography–computed tomography (PET‐CT) demonstrated a 6–7 cm cervical mass extending into the proximal two‐thirds of the vagina, with indistinct margins between the lesion and the cervical stroma (Figure 1).

**FIGURE 1:**
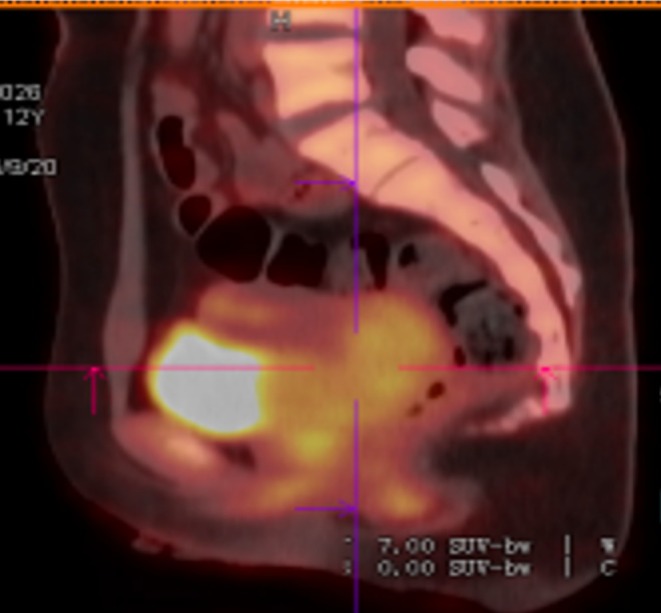
Positron emission tomography–computed tomography (PET‐CT) revealed a 6–7 cm cervical mass extending into the proximal two‐thirds of the vagina, with ill‐defined margins between the lesion and the cervical stroma.

## 3 Differential Diagnosis

A vaginoscopy was first performed, which confirmed that the tumor originated from the cervix (Figure 2A,B), while the vaginal fornix remained uninvolved (Figure 2C,D). Intraoperative findings revealed that the uterus, fallopian tubes, and ovaries appeared grossly normal (Figure 2E,F). The initial differential diagnoses included cervical myoma or polyp. Combined with immunohistochemical profiling, the cervical lesion was ultimately diagnosed as malignant Müllerian papilloma with moderately to poorly differentiated intrauterine adenocarcinoma formation. Immunohistochemistry demonstrated the following profile: CK7 (+), PAX‐8 (+++), PR (+), ER (−), Vim (−), CD99 (+++), Pax‐2 (+), PTEN (+), EMA (+), P53 (+), P16 (+), HER‐2 (+), GATA3 (−), MLH1 (+), PMS2 (+), MSH2 (+), MSH6 (+), and Ki‐67: 45%. According to the 2023 International Federation of Gynecology and Obstetrics (FIGO) revised staging system for cervical cancer, the case was classified as stage IB3.

**FIGURE 2:**
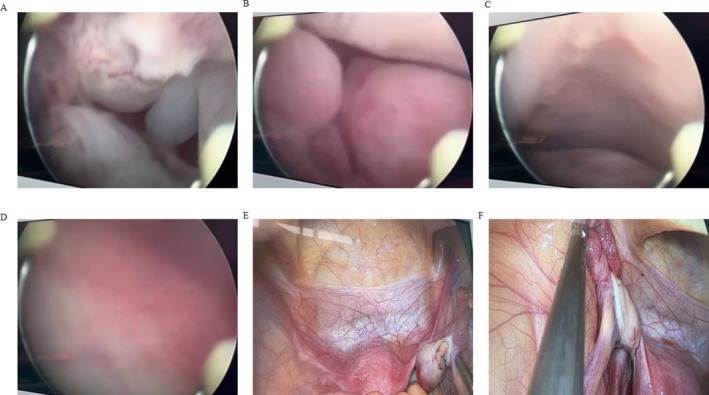
Intraoperative findings confirmed that the tumor originated from the cervix (A, B), while the vaginal fornix was uninvolved (C, D). The uterus, fallopian tubes, and ovaries appeared grossly normal (E, F).

## 4 Conclusion and Results

The surgical procedure consisted of radical hysterectomy, bilateral salpingo‐oophorectomy, pelvic lymph node dissection, and para‐aortic lymph node sampling. Intraoperative findings revealed a 6–7 cm friable, necrotic‐appearing mass arising from the cervix, with no involvement of the vaginal fornix (Figure 3A,B). Pathological examination confirmed cervical malignant Müllerian papilloma with moderately to poorly differentiated intrauterine adenocarcinoma formation (Figure 4A). The tumor invaded approximately one‐third of the cervical stromal depth; however, the surgical margins, vaginal fornix, ovaries, and fallopian tubes were all negative for tumor involvement. All resected lymph nodes were negative for metastasis.

**FIGURE 3:**
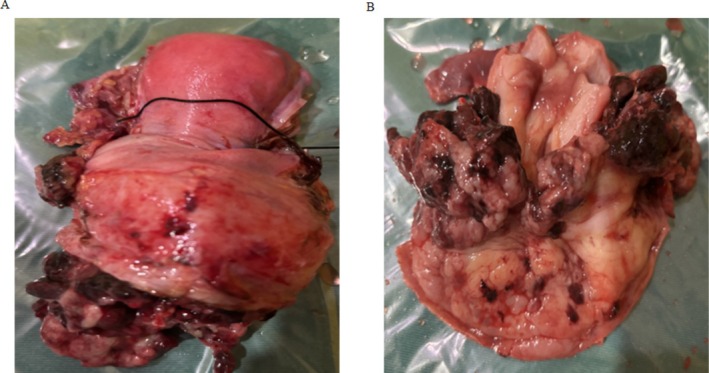
Examination of the surgical specimen revealed a 6–7 cm cervical mass with a friable, necrotic‐appearing consistency. The vaginal fornix was uninvolved (A, B).

**FIGURE 4:**
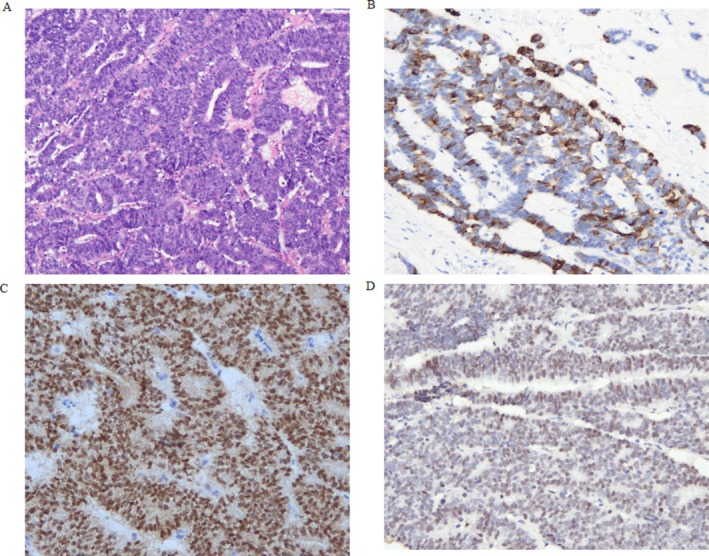
Pathological examination confirmed cervical malignant Müllerian papilloma with moderately to poorly differentiated intrauterine adenocarcinoma formation. (A) Hematoxylin and eosin (HE) staining, ×100; (B) Tumor cells showing positive immunoreactivity for CK7 (+), ×100; (C) Tumor cells showing strong positive immunoreactivity for PAX‐8 (+++), ×100; (D) Tumor cells showing positive immunoreactivity for progesterone receptor (PR) (+), ×100.

Immunohistochemical profiling was consistent with the initial biopsy findings, showing: CK7 (+) (Figure 4B), PAX‐8 (+++) (Figure 4C), and PR (+) (Figure 4D). Postoperatively, given the large tumor size and high‐grade histology, the patient received adjuvant therapy and underwent close follow‐up every three months. At one‐year follow‐up, the patient showed no clinical or radiological evidence of recurrence.

## 5 Discussion

Müllerian papillomas arising from the vagina or uterine cervix have rarely been reported in children and young girls. These tumors are typically benign and commonly present with vaginal bleeding [2]. Due to the paucity of relevant reports, literature on cervical Müllerian papilloma remains sparse.

Accurate diagnosis requires examination under anesthesia and vaginoscopy with biopsy. In children presenting with vaginal bleeding or discharge—particularly those with ultrasound findings suggestive of cervicovaginal abnormalities—vaginoscopy under general anesthesia is essential. For benign Müllerian papillomas, treatment consists of complete local excision. Although local recurrences are not uncommon, the prognosis for both primary and recurrent disease remains excellent [3].

However, some studies suggest that benign Müllerian papillomas may recur and, in rare instances, develop borderline or overtly malignant features if the original tumor was incompletely excised [4]. For malignant Müllerian papillomas, most experts recommend hysterectomy. The literature indicates a high recurrence rate following surgical management, while chemotherapy and radiation therapy have not been routinely employed in the absence of extensive pelvic or residual disease [5]. Poor prognostic factors include depth of stromal invasion and high‐grade malignant features. Long‐term follow‐up of these patients is therefore recommended.

Nevertheless, reports of cervical malignant Müllerian papilloma in children remain exceedingly rare. Due to the scarcity of this tumor in the pediatric population, optimal management strategies have yet to be established. In the present case, we describe a 12‐year‐old child who presented with vaginal discharge. PET‐CT revealed a 6–7 cm cervical mass protruding into the proximal two‐thirds of the vagina, with ill‐defined borders between the lesion and the cervical stroma. To determine disease stage and guide further treatment, the patient underwent vaginoscopy under general anesthesia. Intraoperative findings confirmed that the tumor originated from the cervix, while the vaginal fornix was uninvolved. The disease was classified as stage IB3 according to the International Federation of Gynecology and Obstetrics (FIGO) staging system, and surgical intervention was accordingly pursued.

Intraoperatively, the uterus, fallopian tubes, and ovaries appeared grossly normal. The procedure included radical hysterectomy, bilateral salpingo‐oophorectomy, pelvic lymph node dissection, and para‐aortic lymph node sampling. Pathological examination confirmed cervical malignant Müllerian papilloma with moderately to poorly differentiated intrauterine adenocarcinoma formation. The tumor invaded approximately one‐third of the cervical stroma; however, surgical margins, vaginal fornix, ovaries, and fallopian tubes were negative for malignancy. All resected lymph nodes were free of metastasis. given the large tumor size and high‐grade histology, the patient received adjuvant therapy.

This case underscores the importance of accurate clinical assessment and precise histological characterization to prevent misdiagnosis and subsequent inappropriate treatment. In summary, cervical malignant Müllerian papilloma in children is a rare and diagnostically challenging tumor that may present with nonspecific symptoms such as vaginal discharge or bleeding. Surgical resection remains fundamental to preventing recurrence, and long‐term follow‐up is mandatory.

## Author Contributions

**Na Guo:** conceptualization, investigation, methodology, writing – original draft. **Hui Liu:** writing – review and editing.

## Funding

The authors declare that no funds, grants, or other support were received during the preparation of this manuscript.

## Consent

A written consent was obtained from the parents of the patient for the publication of the case and images.

## Conflicts of Interest

The authors declare no conflicts of interest.

## Data Availability

The data that support the findings of this study are available from the corresponding author upon reasonable request.
